# Development of a Quality Measure to Improve HIV and Syphilis Screening in the Emergency Department: A Modified Delphi Approach

**DOI:** 10.1016/j.acepjo.2025.100203

**Published:** 2025-07-03

**Authors:** Tehreem Rehman, Christopher Bennett, Herbert C. Duber, Michael S. Lyons, Michael P. Phelan, Rachel E. Solnick, Kimberly A. Stanford, Erena Weathers, Arjun K. Venkatesh, Michelle P. Lin

**Affiliations:** 1 Department of Emergency Medicine, Mount Sinai Hospital, New York, New York, USA; 2 Department of Emergency Medicine, Stanford University, Stanford, California, USA; 3 Department of Emergency Medicine, University of Washington School of Medicine, Seattle, Washington, USA; 4 Department of Emergency Medicine, Ohio State University Wexner Medical Center, Columbus, Ohio, USA; 5 Center for Emergency Medicine, Cleveland Clinic, Cleveland, Ohio, USA; 6 Department of Emergency Medicine, Icahn School of Medicine at Mount Sinai, New York, New York, USA; 7 Section of Emergency Medicine, University of Chicago, Chicago, Illinois, USA; 8 Department of Emergency Medicine, NYC Health + Hospital/Elmhurst, New York, New York, USA; 9 Department of Emergency Medicine, Yale School of Medicine, New Haven, Connecticut, USA

**Keywords:** social emergency medicine, reproductive health, quality screening, infectious disease

## Abstract

Emergency department (ED) screening is an important strategy to address the persistent human immunodefiency virus (HIV) epidemic; however, ED screening rates remain low. This study describes the development of a quality measure to improve screening rates for HIV among ED patients. A panel of 10 emergency physicians with expertise in ED-based preventive health and quality measurement employed a 3-round modified Delphi process. The panel reviewed peer-reviewed literature and national guidelines to identify high-risk patient populations; participated in anonymous surveys to rank ED screening approaches based on the impact and feasibility of measurement and practice; and discussed survey rankings in consensus discussions. The highest impact and most feasible group to target for quality measurement were ED patients already undergoing testing for other sexually transmitted infections (ie, “co-screening”). This study successfully designed a quality measure to improve coscreening for HIV in the ED. Implementing this measure could enhance detection and subsequent linkage to care, ultimately contributing to the control of these epidemics. The focus on combining screening for HIV with diagnostic testing for patients with signs and symptoms of sexually transmitted infection aligns with an emerging approach in health care settings that balances expected health impact with feasibility challenges.

**The Bottom Line**

This study developed a quality measure to improve human immunodefiency virus (HIV) screening in the Emergency Department (ED) using a modified Delphi process with 10 expert emergency physicians. The panel identified coscreening for HIV in patients undergoing testing for other sexually transmitted infections (STIs) as the most feasible approach. Implementing this measure could enhance HIV detection and linkage to care among safety-net populations and bolster the role of US EDs in improving population health.

## 1 Introduction

Approximately 13% of the 1.2 million people in the United States with human immunodefiency virus (HIV) are unaware of their diagnosis.^1^ Early diagnosis and treatment can reduce mortality and morbidity for individuals with HIV and syphilis and, by reducing transmission, improve population health.^2^^,^^3^ As part of the safety net, the emergency department (ED) can play a pivotal role in population health—particularly for patients who experience barriers to accessing other health care.^4^ The Centers for Disease Control and Prevention (CDC) recommends routine, opt-out HIV screening for individuals aged 13 to 64 years in all health care settings.^5^ Nonetheless, ED-based HIV testing rates have stagnated at 0.8% in 2020.^6^

One strategy to improve low adherence to guidelines is to develop and implement a quality measure with the aim of improving HIV screening rates. Although clinical guidelines provide flexible, evidence-based recommendations for best practices, quality measures are specific, quantifiable tools used to hold providers accountable for health care structures, processes, and outcomes (Fig). Quality measurement standardizes data collection, enabling better tracking of screening rates to guide improvements in care. Quality measures can also create incentives for practice change when utilized for benchmarking and accountability in pay-for-performance programs.

**Figure 1:**
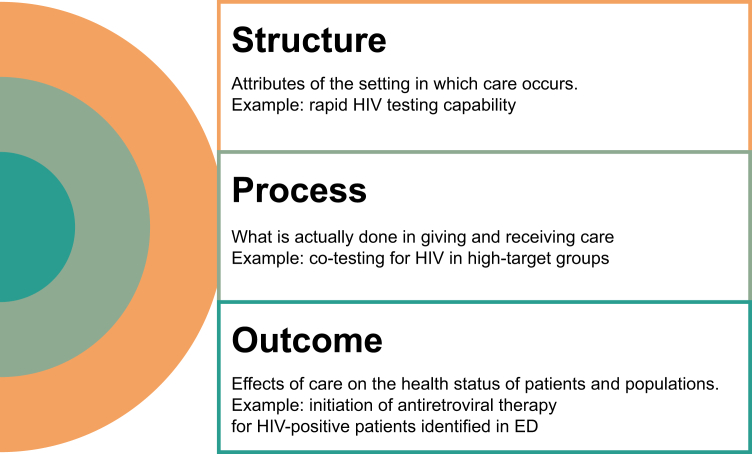
Types of quality measures. ED, emergency department.

Given the potential for ED screening to close major public health gaps and evidence demonstrating persistently low ED screening rates, the American College of Emergency Physicians (ACEP) convened an expert panel to develop a quality measure to improve ED screening rates for HIV testing. The purpose of this manuscript was to describe the development of a consensus-based quality measure to improve the rates of ED HIV screening.

## 2 Methods

### 2.1 Selection of Expert Panel

Panelists were selected through recommendations from the ACEP Quality and Patient Safety Committee, the Public Health Committee, and the Diversity, Inclusion, and Health Equity Section, including the chair of each Committee/Section, and through snowball sampling via recommended experts. The panel comprised 10 emergency physicians with expertise in ED-based preventive health screening and/or quality measurement.

### 2.2 Identifying Appropriate ED Patients for Coscreening

The expert panel convened to discuss the high-impact patient populations to maximize effectiveness while considering the resource use associated with ED-based screening and the feasibility of measurement. Panelists reviewed existing peer-reviewed literature and federal agency publications to identify patient populations with a high likelihood of exposure to or infection with undetected HIV or for whom the consequences of infection are particularly dire (eg, pregnant individuals). Target populations were included in a preliminary list of additional patient groups to include for targeted HIV testing in the ED. Table 1 lists the groups considered for prioritization for HIV screening. Panelists also considered the Denver HIV Risk Score. This validated HIV risk prediction tool does not correspond to a specific subgroup but has been shown to identify individuals at increased risk for HIV based on demographic data.^7^ Table 2 lists select publications with grading classifications that were included in the literature review presented to panelists at the first meeting.^8^, ^9^, ^11^, ^12^, ^13^, ^14^, ^15^, ^16^, ^17^, ^18^, ^19^, ^20^, ^21^, ^22^, ^23^, ^24^, ^25^

**Table 1:** Modified Delphi process ranking scores (in decreasing order of round 2 scores) and final consensus.

Inclusion group (listed in descending order based on round 2 scores)	Round 1 (mean ± SD)		Round 2 (mean ± SD)	Round 3 (inclusion in final list)
	Impact	Feasibility	Combined (impact + feasibility)	
Testing or diagnosis for syphilis			5.00 ± 0.00	Yes
Any recent STI			4.80 ± 0.40	No
High community prevalence			4.00 ± 1.26	No
People who inject drugs	4.75 ± 0.66	3.50 ± 1.22	4.00 ± 1.10	No
Patients with a positive pregnancy test in the ED			4.00 ± 0.89	No
All patients admitted from ED, regardless of admitting diagnosis			3.40 ± 1.62	No
All pregnant patients regardless of reason for ED visit			3.40 ± 1.02	No
Recently incarcerated persons	3.88 ± 1.45	3.00 ± 1.51	3.00 ± 1.67	No
Persons in correctional facilities	3.88 ± 1.27	3.00 ± 1.41	3.00 ± 1.67	No
Victims of sexual assault	4.25 ± 0.97	3.86 ± 0.99	3.00 ± 1.67	No
Unprotected intercourse with partners of unknown status			3.00 ± 1.67	No
Men who have intercourse with men	4.88 ± 0.33	3.00 ± 1.66	3.00 ± 1.41	No
Intercourse workers	4.88 ± 0.33	3.00 ± 1.60	2.60 ± 1.36	No
Transactional intercourse (encompassing but not limited to intercourse workers)			2.40 ± 1.50	No
Patient with mental health comorbidities			2.20 ± 1.17	No
Testing or diagnosis for gonorrhea/chlamydia	4.63 ± 0.70	5.00 ± 0.00		Yes
Pregnant patients	4.13 ± 1.05	4.71 ± 0.45		No
Trauma patients	2.75 ± 1.48	3.43 ± 1.59		No
Denver HIV Risk Score	4.00 ± 1.32	3.29 ± 1.75		No
Transgender women	4.63 ± 0.70	2.71 ± 1.58		No

ED, emergency department; STI, sexually transmitted infections.

**Table 2:** Summary of select literature reviewed by expert Panel.

Title (y)	Target group	Summary of evidence
Cotesting for Human Immunodeficiency Virus and Sexually Transmitted Infections in the Emergency Department (2022)^8^	Diagnosis or treatment for another STI	The study identified over 13 million high-risk encounters where gonorrhea and chlamydia testing were performed, revealing that HIV screening occurred in only 3.9% of these visits, and syphilis testing in 2.9%.
Routine, Opt-Out, Emergency Department Syphilis Testing Increases HIV Preexposure Prophylaxis Uptake (2023)^9^	Diagnosis or treatment for another STI	The study found that syphilis diagnosis was associated with increased perception of HIV risk and increased initiation of PrEP.
Multidisciplinary Approach to Improve Human Immunodeficiency Virus and Syphilis Testing Rates in Emergency Departments (2022)^10^	Diagnosis or treatment for another STI	The study implemented a series of interventions—including rapid HIV testing, a standardized STI-screening order panel, and clinician feedback—which collectively increased HIV testing from 5% to 36% and syphilis testing from 9% to 39%.
HIV and Injection Drug Use (2017)^11^	PWID	The report highlights that sharing needles and other injection equipment significantly increases the risk of HIV infection among PWID. It also emphasizes the effectiveness of harm reduction strategies, such as syringe services programs and access to substance use disorder treatments, in reducing HIV transmission rates among this population.
HIV Surveillance Report: Diagnoses of HIV Infection in the United States and Dependent Areas (2022)^12^	PWID	The report entails a comprehensive analysis of national HIV surveillance data collected through June 2018, offering a robust and detailed overview of HIV infection diagnoses across various demographics and regions. The report presents data on trends over time, modes of transmission, and disparities among different populations, serving as a critical resource for public health planning and policy development.
HIV detection by an emergency department HIV screening program during a regional outbreak among people who inject drugs (2021)^13^	PWID	The study found that the annual number of HIV diagnoses made by the emergency department increased from 20 to 42 overall, and from 1 to 18 among PWID after implementation of an urban emergency department’s HIV screening program.
Men who have intercourse with men (2021)^14^	MSM	The report highlights that MSM are disproportionately at risk for HIV infection, with an estimated lifetime risk of 1 in 6, compared to one in 524 for heterosexual men and one in 253 for heterosexual women.
Diagnosing HIV in Men Who Have intercourse with Men: An Emergency Department’s Experience (2012)^15^	MSM	This study entailed a retrospective analysis of an emergency department-based HIV screening program, which identified that MSM were 10 times more likely than non-MSM to have undiagnosed HIV infections.
Prevalence of HIV Among US Female intercourse Workers: Systematic Review and Meta-analysis (2016)^16^	Intercourse workers	The study includes a systematic review and meta-analysis of 14 studies conducted between 1987 and 2013, encompassing a total of 3975 adult female intercourse workers in the United States. The pooled estimate of HIV prevalence was 17.3% (95% CI: 13.5%-21.9%).
HIV Testing and Counseling Among Female intercourse Workers: A Systematic Literature Review (2018)^17^	Intercourse workers	This study is a systematic review of 36 articles published between January 2000 and November 2017, which identified financial and time costs, as well as stigma and discrimination, as significant barriers to HIV testing among female intercourse workers.
HIV in Prisons, 2001-2010 (2012)^18^	Persons in correctional facilities	This is a comprehensive analysis of national data collected through the Bureau of Justice Statistics’ National Prisoner Statistics Program and Deaths in Custody Reporting Program. The report indicates a decline in the rate of HIV/AIDS among state and federal prison inmates from 194 cases per 10,000 inmates in 2001 to 146 per 10,000 at yearend 2010.
Understanding the Health Needs of Incarcerated Men Living with HIV/AIDS: A Primary Health Care Approach (2011)^19^	Persons in correctional facilities	The article identifies significant barriers to effective HIV care in correctional settings, including stigma, discrimination, and limited access to healthcare services.
HIV among persons incarcerated in the USA: a review of evolving concepts in testing, treatment, and linkage to community care (2013)^20^	Persons in correctional facilities	This is a comprehensive analysis of national data and peer-reviewed studies, offering a robust understanding of HIV prevalence, testing strategies, treatment protocols, and linkage to community care for incarcerated individuals in the United States. The review highlights that the HIV seroprevalence among incarcerated individuals is approximately 1.5%, which is 3 times higher than that of the general US population.
Studies show new progress in HIV testing in emergency departments (2011)^21^	Pregnant women	This report examined the implementation and outcomes of routine HIV testing in EDs across the United States. The findings indicate that while the number of EDs offering HIV testing increased substantially between 2004 and 2009, significant challenges remain in integrating routine, opt-out HIV testing into standard ED protocols.
HIV testing among pregnant women with prenatal care in the United States: An analysis of the 2011-2017 National Survey of Family Growth (2020)^22^	Pregnant women	The study analyzed data from the National Survey of Family Growth between 2011 and 2017, encompassing a nationally representative sample of pregnant women receiving prenatal care in the United States. It found that approximately 25% of these women reported not being tested for HIV during their pregnancy.
High Rate of HIV Among Trauma Patients Participating in Routine Emergency Department Screening (2023)^23^	Trauma patients	This study entailed a retrospective cross-sectional design, which analyzed 147,430 emergency department encounters at a Level 1 trauma center from May 1, 2018, through May 1, 2021. It found that trauma patients were less likely to be screened for HIV than medical patients (18.1% vs 25.6%) but had higher rates of HIV infection (2.2% vs 1.3%).
HIV Infection, Risk, Prevention, and Testing Behaviors Among Transgender Women—National HIV Behavioral Surveillance, 7 US Cities, 2019-2020 (2021)^24^	Transgender women	This is a comprehensive analysis of systematically collected data from the National HIV Behavioral Surveillance among transgender women in 7 US cities. The report highlights a disproportionately high HIV prevalence among transgender women, particularly among transgender women of color.
HIV-related care for transgender people: a systematic review of studies from around the world (2019)^25^	Transgender women	This is a comprehensive analysis of 62 peer-reviewed quantitative studies published up to April 2018, encompassing various aspects of HIV-related care for transgender populations, including PrEP, postexposure prophylaxis, HIV testing, healthcare access, and treatment adherence. The review highlights significant barriers faced by transgender individuals in accessing HIV-related care, such as stigma, discrimination, and a lack of culturally competent healthcare services.

MSM, men who have sex with men; PrEP, preexposure prophylaxis; PWID, people who inject drugs; STI, sexually transmitted infections.

### 2.3 Modified Delphi Method

A modified Delphi approach, a structured consensus building technique, was utilized to achieve agreement among expert panelists on groups to prioritize ED HIV testing.^26^ The modified Delphi process is a systematic approach developed by RAND/UCLA in the 1950s for integrating expert opinions and evidence through multiple rounds of rating and discussion.^27^ This approach has been successfully used to develop quality improvement interventions and medical guidelines and has been shown to have high predictive validity compared with subsequent randomized trials.^28^ We performed 3 iterative Delphi rounds between July and September 2023, which entailed surveys distributed through Qualtrics XM software to rank the inclusion and exclusion criteria for the quality measures. Questionnaire responses were shared anonymously to avoid bias and influence from dominant voices. Each round’s survey was followed by an interactive meeting with all panelists to facilitate a robust discussion on variability in participant rankings and directly address any problematic assumptions or limitations to achieve consensus criteria. This quality improvement project was deemed exempt from institutional review board review.

### 2.4 Round 1

The first modified Delphi meeting introduced panelists to the modified Delphi process. It provided a brief overview of the peer-reviewed literature and policy recommendations regarding high-target groups to prioritize for HIV screening. The subsequent survey (Supplementary Appendix 1) asked panelists to separately rank each high-target category for HIV testing based on a Likert scale for impact (1 = lowest impact to 5 = highest impact) in 2 key domains corresponding to National Quality Forum Quality Measure Evaluation Criteria: (1) importance to measure and report and (2) feasibility and use.^29^ The importance of measuring and reporting was defined as scientific evidence demonstrating a gap in ED testing for each subgroup and evidence demonstrating that ED testing improves health outcomes. Feasibility was defined as (1) the ability to capture risk factors reliably and consistently in the electronic health record; (2) evidence demonstrating quality improvement efforts can improve testing rates, and (3) cost and length of stay associated with additional testing, including phlebotomy. Expert panelists ranked each group on both domains for HIV testing. Finally, panelists identified any high-target groups that needed to be added to the initial proposed groups.

### 2.5 Round 2

The first-round ranking survey results were shared and discussed with panelists in the second round of the modified Delphi method. The second meeting introduced exclusion criteria for consideration (conditions for which HIV testing would not be appropriate, eg, known HIV) and refined ED-specific inclusion criteria. It clarified the scope of other groups to consider, such as the testing time frame for other “recent sexually transmitted infections (STIs).”

A second survey (Supplementary Appendix 2) was distributed to expert panelists. Panelists reranked impact and feasibility when applying the Likert scale to rank an expanded list of inclusion groups, including suggestions from panelist responses to the first-round survey. Panelists specified which STIs should be included in the proposed category “any recent STI test or diagnosis” (eg, chlamydia, trichomoniasis, etc). Expert panelists also selected an evidence-based option for a time frame to apply to the proposed exclusion criteria of a recent negative HIV test.

### 2.6 Round 3

During the third and final modified Delphi meeting, results from the second survey were shared for discussion with panelists. The last meeting finalized the inclusion and exclusion criteria for HIV screening. After the meeting, expert panelists completed a third and final survey (Supplementary Appendix 3) to achieve consensus on the highest impact groups for the HIV screening measure. The third survey also finalized exclusion criteria for recent negative HIV tests.

Ultimately, we adhered to the ACCORD checklist with respective to iterative survey design, dissemination while preserving anonymity, the Delphi method for consensus building, and alignment of recommendations with preexisting literature.

## 3 Results

### 3.1 Round 1

Round 1 survey had a 100% participation rate. Expert panelists separately ranked the impact and feasibility of including groups for HIV screening, with mean ranking scores listed in Table 1. Inclusion groups ranked as both high impact and feasible (>4 mean score for both) were diagnosis or treatment for gonorrhea/chlamydia (cotesting) and pregnant patients. The inclusion groups ranked as low impact and/or infeasible (<3 mean score for either one) were trauma patients and transgender women. The remaining groups were classified as intermediate impacts due to concerns about feasibility despite moderate or high importance to measure.

Expert panelists proposed the addition of 2 inclusion groups with high community prevalence and patients with mental health comorbidities. Specifically for the HIV screening measure, panelists recommended further inclusion of groups of any recent STI, cotesting for syphilis, not just gonorrhea and chlamydia (GC), transactional intercourse (encompassing but not limited to intercourse workers), and condom-less intercourse with a partner of unknown status.

### 3.2 Round 2

During the second meeting, expert panelists discussed groups with the highest potential impact from screening, such as patients being cotested for other STIs. Panelists reached a consensus on excluding low-impact or infeasible groups (eg, trauma patients and Denver HIV Risk Score). Panelists engaged in a robust discussion on the intermediate category groups and new suggested groups to refine the inclusion criteria overall. The expert panel recognized that CDC recommendations for STI testing after sexual assault are currently to be considered on an individual basis due to a lack of data on STI rates among sexual assault patients. Panelists also debated how to exclude patients who were recently screened outpatient or plan to be screened outpatient (eg, a pregnant patient with a recent or upcoming obstetrics ambulatory clinic appointment). Several panelists agreed that redundant screening of those patients in the ED may not be an appropriate use of resources and that patients with a new ED diagnosis of pregnancy (as confirmed by a positive pregnancy test) should be considered differently from all pregnant ED patients.

The round 2 survey had a 90% participation rate, with ranking scores in Table 1. For HIV screening, high impact and feasibility inclusion groups with a mean rank score of 4 or greater for impact and feasibility were cotested for syphilis, any recent STI, patients with a positive pregnancy test in the ED, people who inject drugs, and high community prevalence. Groups with a mean rank less than 3 for importance and/or feasibility were intercourse workers, transactional intercourse (encompassing but not limited to intercourse workers), and patients with mental health comorbidities.

### 3.3 Round 3

During the third meeting, panelists reached a consensus on prioritizing diagnosis or treatment for GC as a high impact for HIV screening. The round 3 survey had a 100% participation rate. The expert panel did not reach a consensus on trichomoniasis as an inclusion criterion for the HIV screening measure. The panelists who did not vote for trichomoniasis were concerned that adding other STIs would hinder efficient ED-based testing. One of the 10 panelists did not vote for syphilis to be included in the HIV screening measure due to concerns about the increasing complexity of the cotesting measure without a clear benefit. The expert panel did not reach a consensus on sexual assault as an inclusion group for the HIV screening measure. Three panelists endorsed explicitly excluding all sexual assault patients from the HIV testing measures due to CDC recommendations for personalized testing and/or concerns about feasibility. The expert panel did not reach a consensus on a recent negative HIV test as an exclusion criterion due to concerns about missing a critical window for testing during an incubation period or recurrent exposure among high-target patients. In summary, consensus for inclusion criteria in the quality measure was achieved in round 2 for “Testing or diagnosis for gonorrhea/chlamydia” with a mean feasibility score of 5 and in round 3 for “Testing or diagnosis for syphilis” with a mean combined (impact + feasibility) score of 5.

## 4 Limitations

There are several limitations to our study. We considered including nonemergency physicians, such as registered nurses, patients, and nonphysician public health professionals; however, we did not because the intent was to develop a quality measure narrowly tailored to the ED setting—which should be developed by and intended for use by emergency physicians. Additionally, we sought diverse expert panelists to participate in the modified Delphi process. However, nearly all participants were from academic settings, and representation was lacking from the Southern region (Table 3). Nonetheless, our panel included national experts in public health and quality measurement from diverse genders and career stages. Not all expert panelists participated in every survey round, and the characteristics of nonrespondents could not be determined due to the anonymous nature of the surveys. However, most panelists attended each meeting, where robust discussions ensured that all viewpoints were considered. Panelists were aware of each other’s identities during meetings, which may have introduced response and social desirability bias. Blinding panelists during consensus discussions is optional in the modified Delphi process, as it adds complexity without eliminating the potential influence of group dynamics. To mitigate dominant voices and promote independent thinking, we utilized anonymous surveys. Syphilis was originally considered in the modified Delphi process for a separate quality measure that was not ultimately submitted to CMS for approval. Because this was not intended to be a clinical guideline or systematic review, the strength of evidence for including or excluding specific groups was not evaluated. This work was designed for US EDs and may not be generalizable to other settings.

**Table 3:** Characteristics of modified Delphi expert panelists.

Characteristic	N (%)
Median years since residency	8 (IQR 2-19)
Female	5 (50)
Region	
West	3 (30)
Midwest	3 (30)
Northeast	4 (40)

Total N = 10.

## 5 Discussion

We conducted a modified Delphi process to identify groups to prioritize for HIV testing for an emergency medicine quality measure. The group unanimously endorsed a quality measure to improve ED HIV screening among those undergoing testing for other STIs, including syphilis. Most groups were identified as high impact with respect to the need for improved ED screening. However, several of those were considered less feasible to measure and report, due to factors such as inconsistent documentation of social and behavioral risk factors or lack of local prevalence data for geographic targeting.^11^

When evaluating feasibility, panelists considered the resources needed for screening and appropriate referral of patients with positive screening tests. Nontargeted screening for HIV may not be feasible in all ED settings due to resource limitations, including staffing and crowding.^12^ Although testing itself may be reimbursable, preventive health screenings can yield opportunity costs in ED operations through longer lengths of stay or diversion of staff time from providing acute care.^13^ A quality measure focused on ED patients already undergoing testing for other STIs may overcome these feasibility concerns, given that EDs performing screening for other STIs likely have sufficient resources for HIV and syphilis screening and referral. Despite high rates of coinfection, only 3.9% and 2.9% of ED encounters for GC testing include HIV and syphilis cotesting, respectively.^14^, ^15^, ^16^, ^17^ At the same time, recent evidence demonstrates the feasibility of improving ED screening in this population: One ED system successfully operationalized a multidisciplinary initiative to increase HIV cotesting with gonorrhea and chlamydia from less than 1% to 28%.^18^

Panel recommendations were incorporated into a quality measure submitted to the Centers for Medicare and Medicaid services for ED clinicians participating in the Merit-Based Incentive Payment System. In 2023, the CMS approved the quality measure, entitled “ACEP 66: Cotesting for HIV in Emergency Department patients being tested for other STIs (gonorrhea, chlamydia, syphilis, or trichomonas).” The measure is based on each ED clinician group’s percentage of patients aged 18 and older in the ED who are being tested for other STIs (gonorrhea, chlamydia, syphilis, or trichomonas) are also tested for HIV. Patients who decline testing, leave against medical advice, leave without treatment, leave without being seen, die, and who already have HIV are excluded. As with all the Merit-Based Incentive Payment System quality measures, ED clinician groups select which quality measures to report on and are never mandated to report on any single measure.^19^ Furthermore, scoring is based on both relative performance, comparing clinicians with established benchmarks, and improvement, rewarding those who show significant progress over time. ED clinician groups are neither required nor expected to achieve 100% performance to achieve financial incentives associated with quality improvement.

Beyond early diagnosis and improved treatment outcomes, the secondary effects of ED-based HIV screening include increased awareness, reduced stigma, and potential shifts in sexual health behaviors. However, there are potential negative consequences, including false-positive results leading to unnecessary anxiety, concerns regarding confidentiality in busy ED settings, and potential strain on already overburdened ED staff. Ensuring appropriate patient education, streamlined workflows, and confidentiality protections can mitigate these risks.

It is important to note that although higher rates of HIV testing among high-target ED patients may achieve a desirable process metric, increased screening does not necessarily translate into the patient-centered outcome of linkage and appropriate treatment among those who test positive. Twenty percent of all newly diagnosed HIV patients in 2021 did not achieve linkage to care within 1 month of diagnosis.^20^ Populations with high social vulnerability more often face social and structural determinants of health associated with worse health outcomes and are disproportionately impacted by gaps in HIV linkage to care. Future quality measures can build on the HIV screening quality measure to specifically assess for linkage to care, especially for disproportionately vulnerable patient groups.

Expert panelists discussed why prioritizing certain groups based on gender, sexual orientation, or behaviors may increase the stigma associated with screening and subsequently increase barriers to participation. There is increased consensus on departing from risk-based language to identify patients for STI screening, as it can perpetuate stigma and discrimination and lead to missed opportunities for diagnosis. There was substantial discussion among panelists regarding the extent to which a quality measure tied to reimbursement may disadvantage lower-resourced EDs caring for higher-risk populations. Historically marginalized groups most likely to benefit from HIV testing are not only disproportionately likely to be served by safety-net institutions, but they are also more likely to have had adverse experiences with health care that contribute to mistrust and may be more likely to decline HIV testing.^21^ Nonetheless, panelists felt the benefits of establishing national benchmarks to improve current testing rates outweighed these potential unintended consequences.

Facilities with high rates of undiagnosed HIV, limited access to primary care, and a high burden of STIs are likely to have the greatest public health impact from ED-based HIV screening. However, these same facilities—often safety-net and under-resourced hospitals—may face significant barriers, including lack of funding for rapid testing, limited staff capacity, and challenges with linkage to care. Addressing these disparities will require policy-level support, dedicated funding, and integration with community health resources. Ongoing monitoring and evaluation of the impact of ED HIV screening and the quality measure’s impact on equity are needed.

In conclusion, we convened a group of emergency physicians to develop a quality measure to improve ED HIV screening using a modified Delphi consensus process to assess impact and feasibility. The resulting quality measure focuses on improved screening rates among ED patients undergoing testing for other STIs and has been approved by CMS for use in value-based payment programs. Implementing quality measures to improve rates of HIV screening has the potential to improve health outcomes for structurally marginalized groups treated in ED settings and enhance public health.

## Funding and Support

TR reports that this project was supported by AMA/CDC $25,000 grant for implementation of routine HIV screening at University of Colorado from May 2023 to November 2023. CB reports support from 10.13039/100006108NCATS KL2TR003143, 10.13039/100000060NIAID L30AI178800, and NIAID K08AI181642. RES reports support from NIMH K23MH136923-01. KAS reports support from NIAID K23AI166277, Emergency Medicine Foundation, CDC/NACCHO. AKV reports support from Moore Foundation to study acute infections and support from CMS to develop hospital and health system quality measures and rating systems. MPL reports support from NHLBI K23HL143042.

## Conflict of Interest

CB: Prior honorarium from Gilead Sciences for serving on an advisory board. Other authors have affirmed they have no conflicts of interest to declare. MSL: Investigator-initiated project support paid to institution by Gilead Sciences, Inc., Columbus Public Health, and SAMHSA. KAS: Investigator-initiated project support paid to institution by Gilead FOCUS and HRSA.

## Supplemental material

Supplemental Appendices
