# Supplementary material for: Development of a Quality Measure to Improve HIV and Syphilis Screening in the Emergency Department: A Modified Delphi Approach
Source: J Am Coll Emerg Physicians Open. 2025 Jul 3;6(4):100203. doi: 10.1016/j.acepjo.2025.100203 (PMC12272119; doi:10.1016/j.acepjo.2025.100203)
Supplement: Supplemental Appendices [file mmc1.docx]

**Appendix 1.**

Thank you for participating in the Technical Expert Panel to develop a quality measure to improve ED HIV and Syphilis testing. The purpose of this survey is to identify high-risk groups for inclusion in a Clinical Emergency Data Registry quality measure assessing emergency physician group-level performance tied to payment incentives.


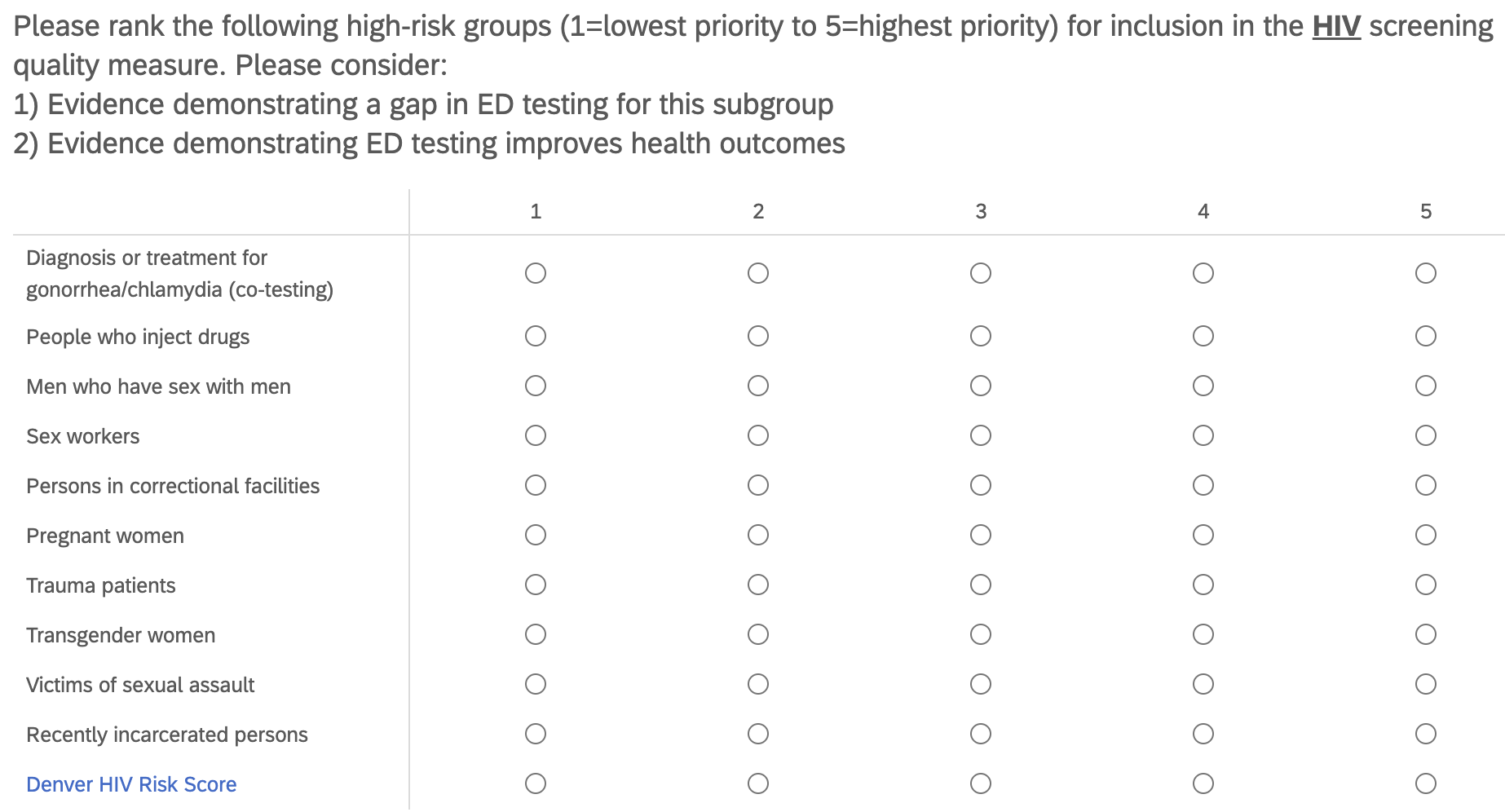


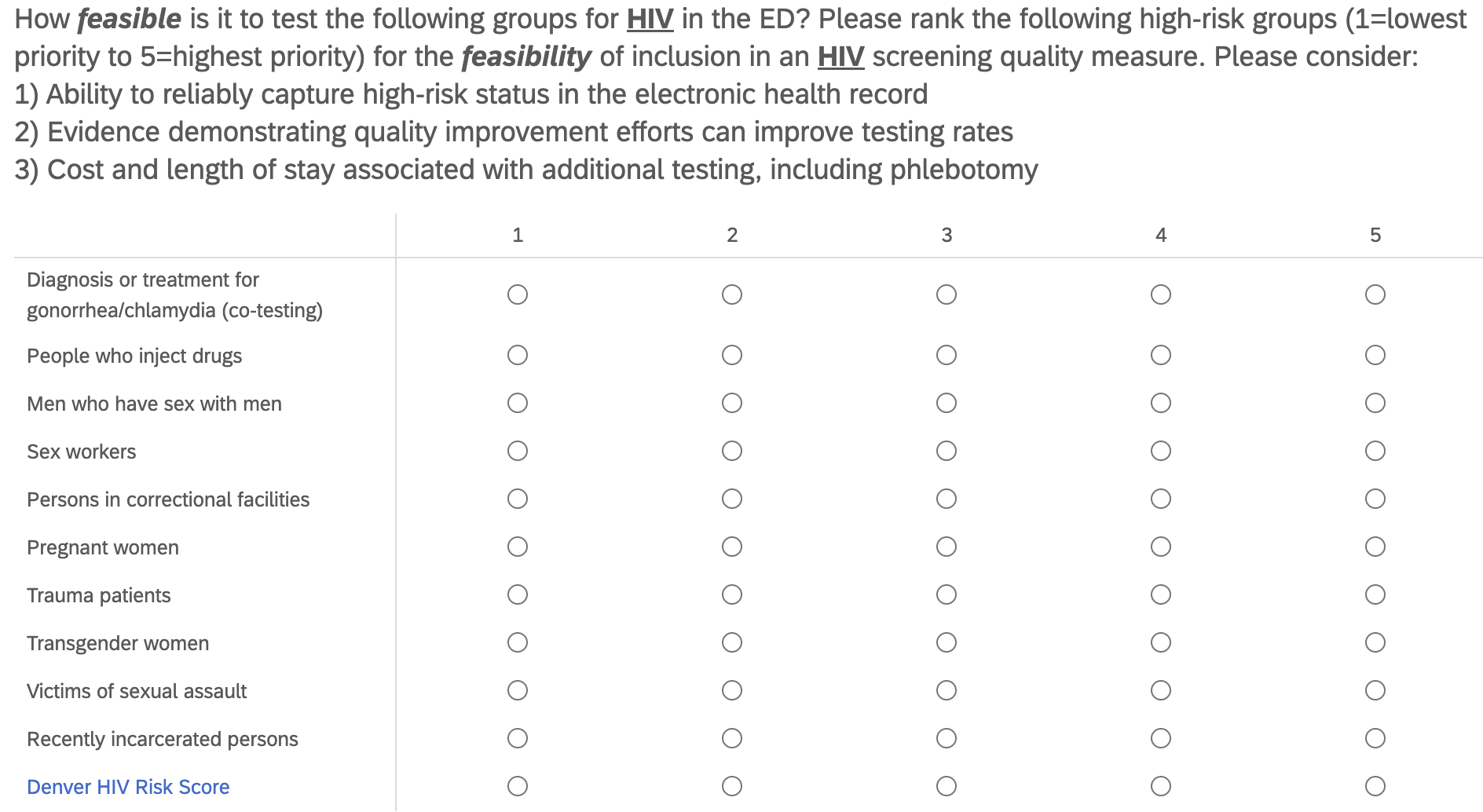


Are there any other high-risk groups for **HIV** testing missing? If so, please list them below.


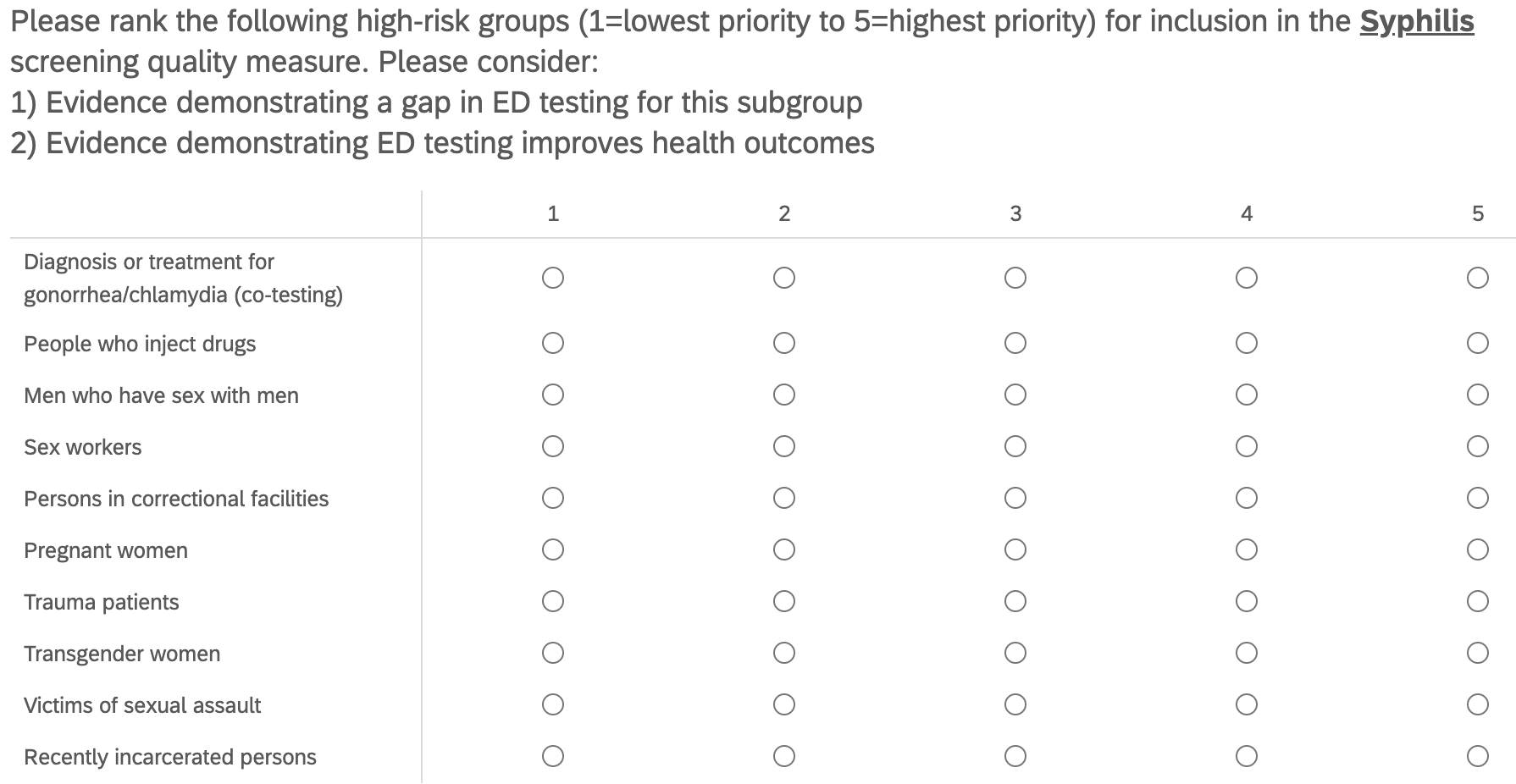


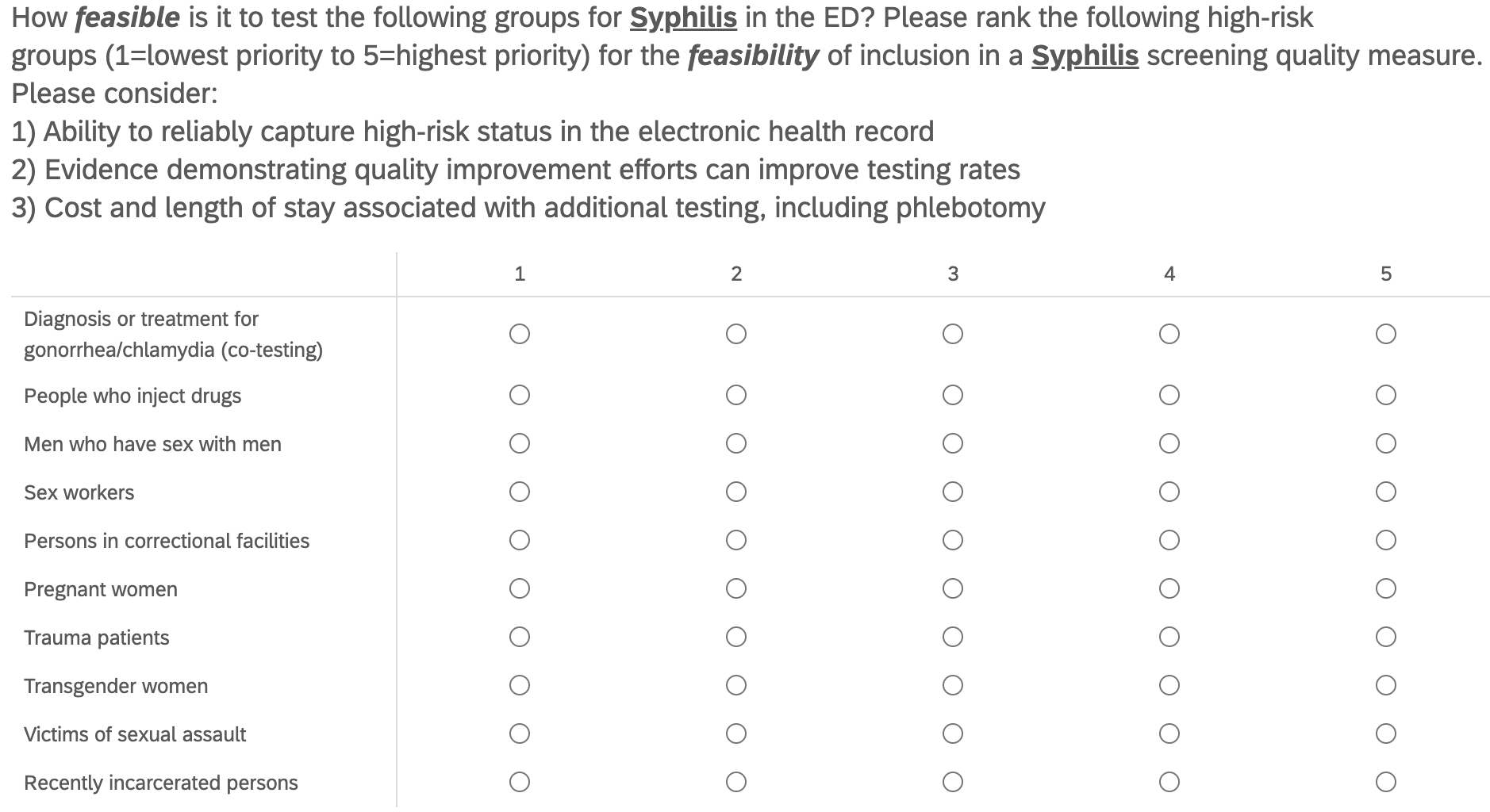


Are there any other high-risk groups for **Syphilis** testing missing? If so, please list them below.

Do you have any general concerns that were not yet addressed? If so, please describe further below.

**Appendix 2.**

Thank you for participating in the Technical Expert Panel to develop a quality measure to improve ED HIV and Syphilis testing.

Based on our last survey results and subsequent meeting, we were able to reach consensus on several groups to include and groups to no longer consider.

The purpose of this survey is to finalize other groups for inclusion (including new ones suggested in the last survey) and consider exclusion criteria in a Clinical Emergency Data Registry quality measure assessing emergency physician group-level performance tied to payment incentives.

**
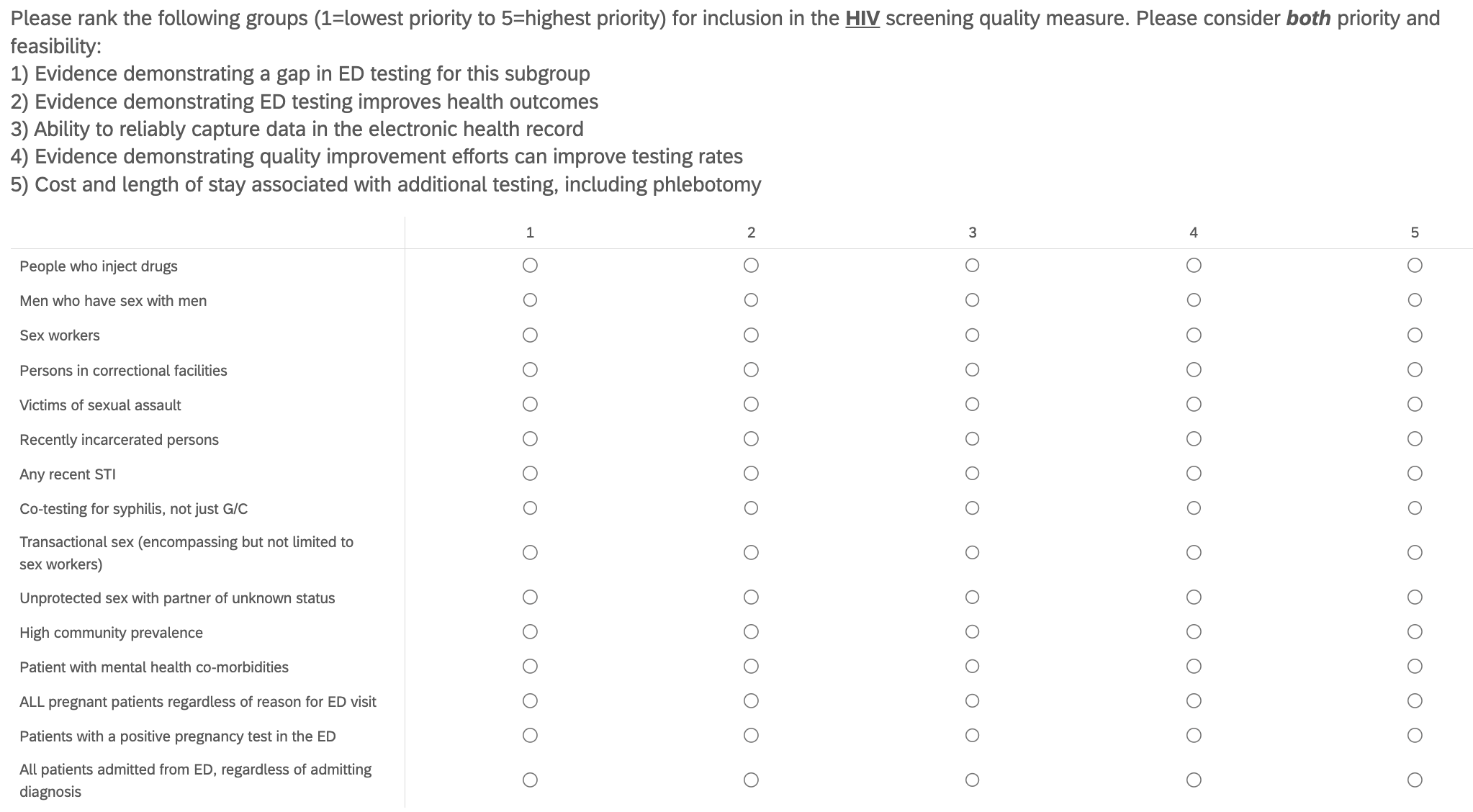
**

**
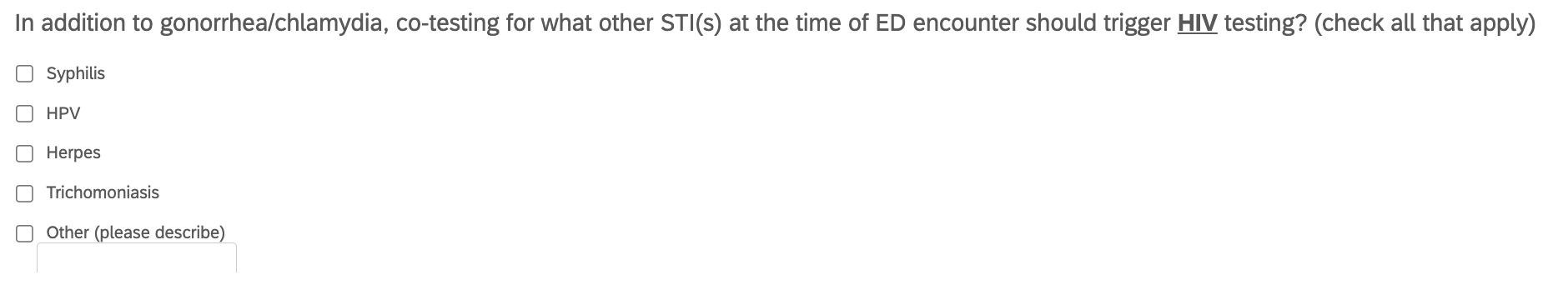
**

**
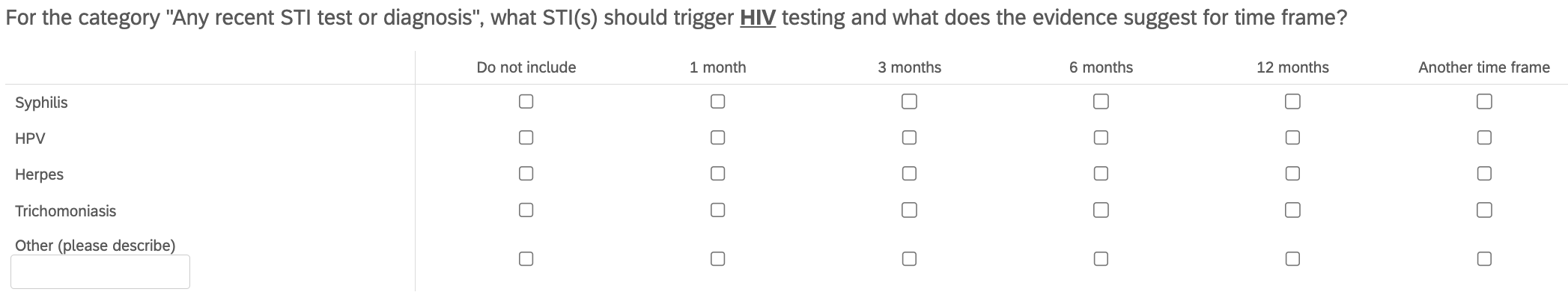
**

**
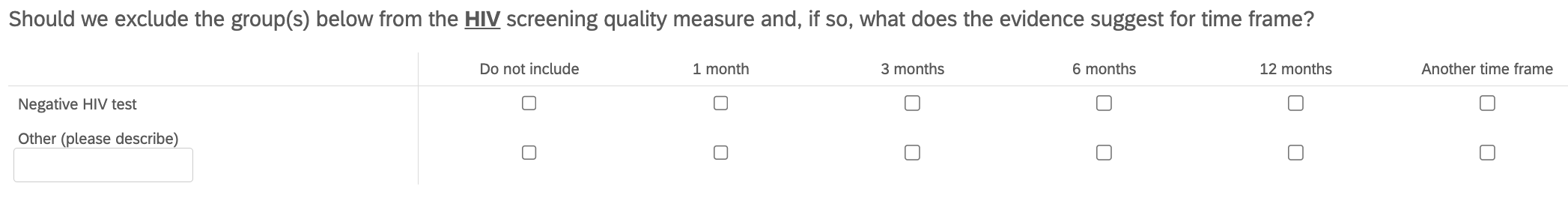
**


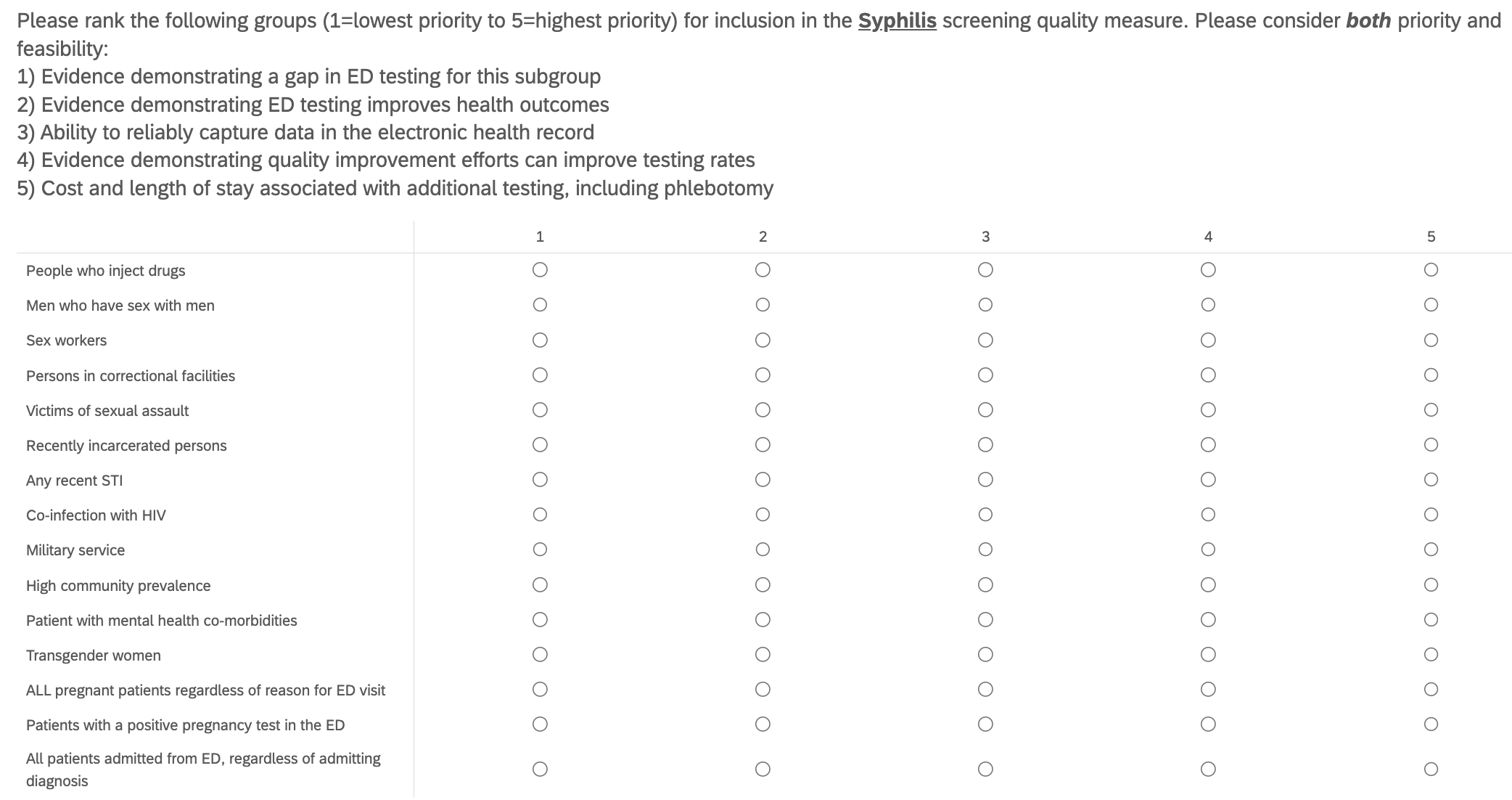


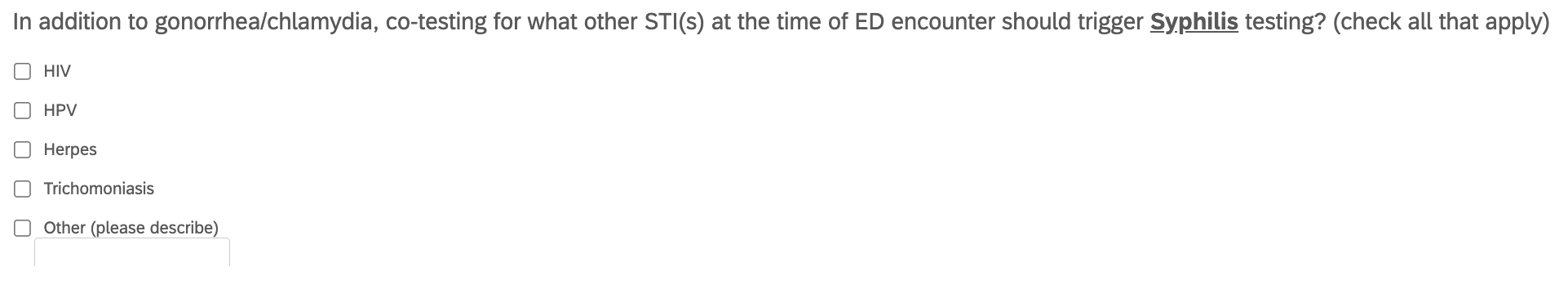


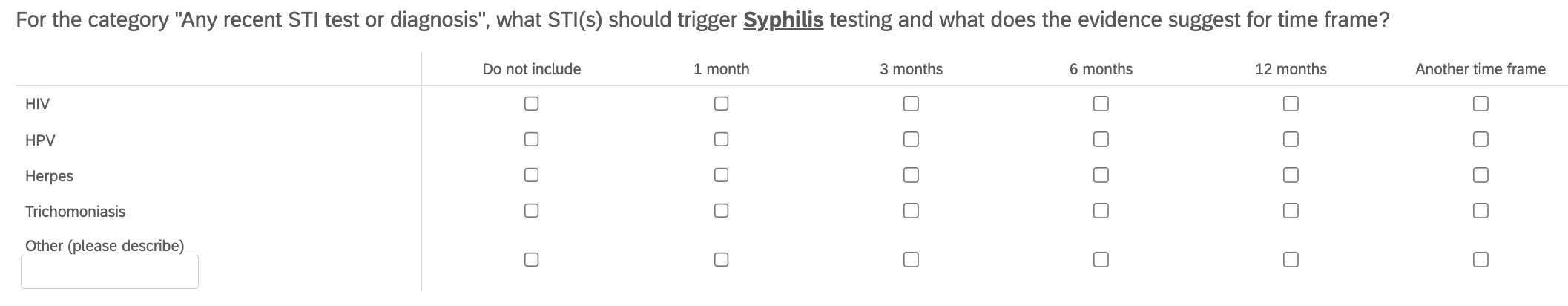


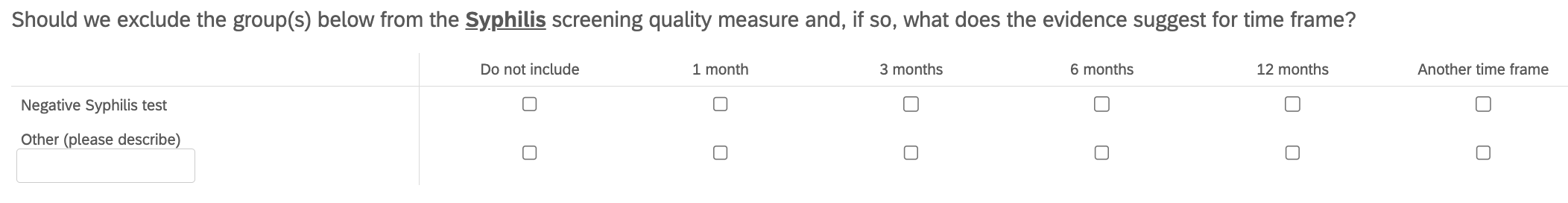


**Appendix 3.**

Thank you for participating in the Technical Expert Panel to develop a quality measure to improve ED HIV and Syphilis testing.

The purpose of this survey is to achieve consensus on final remaining items.

**
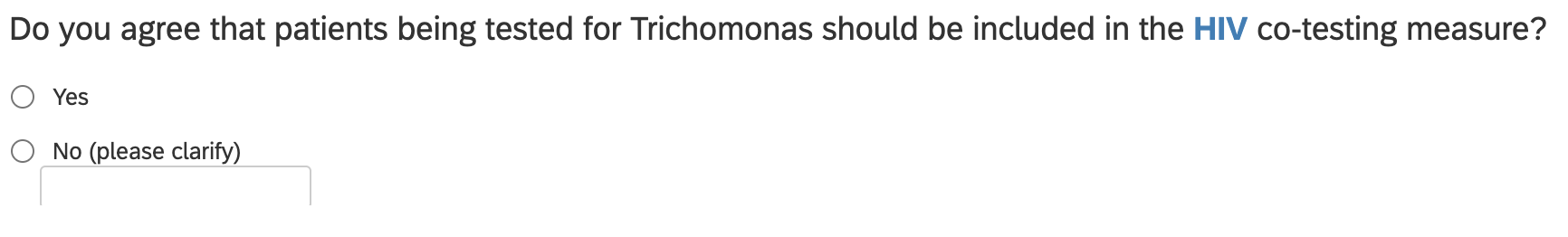
**

**
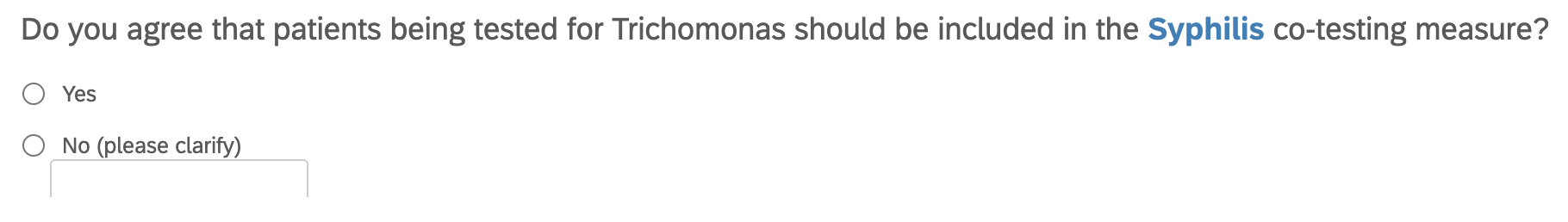
**


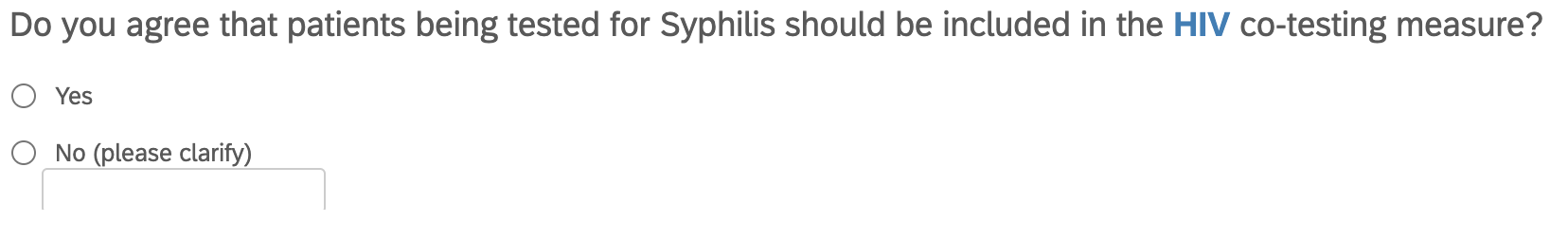


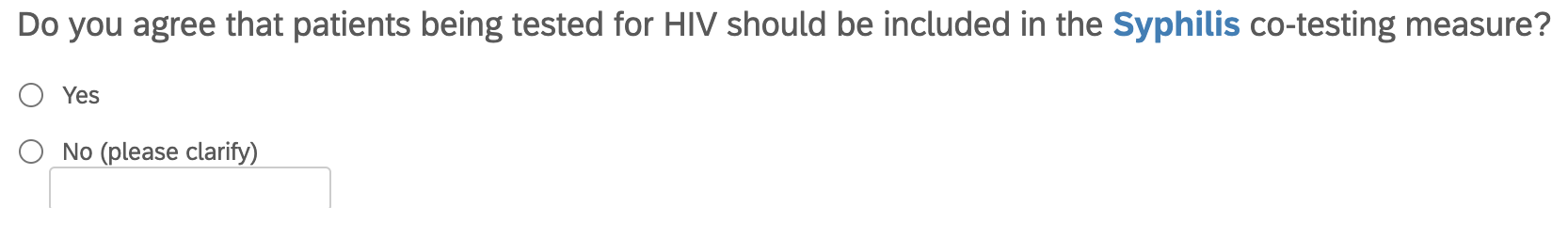


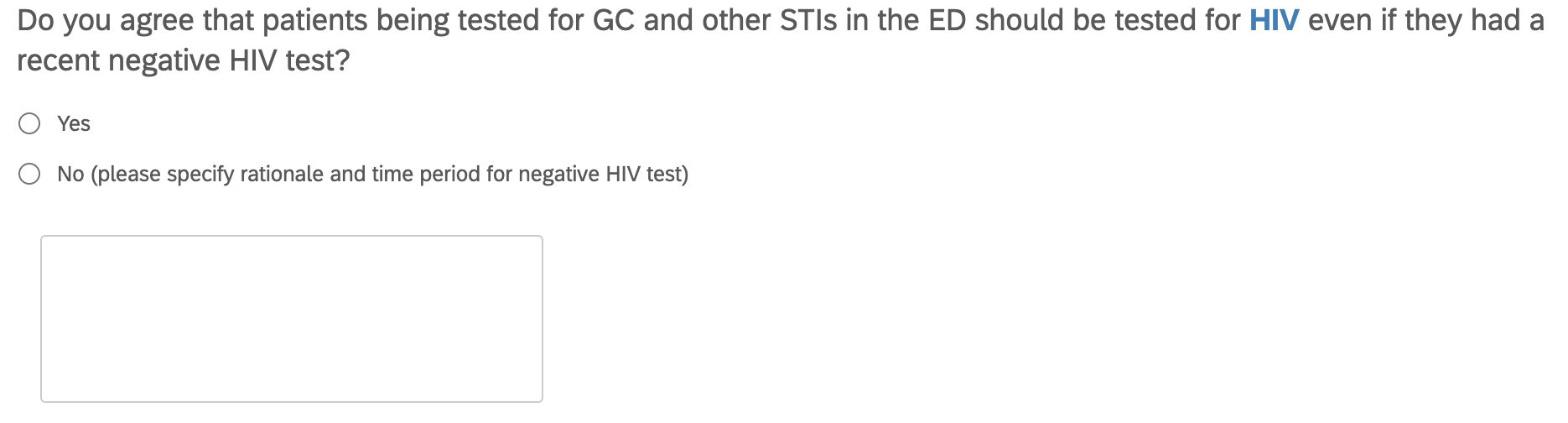


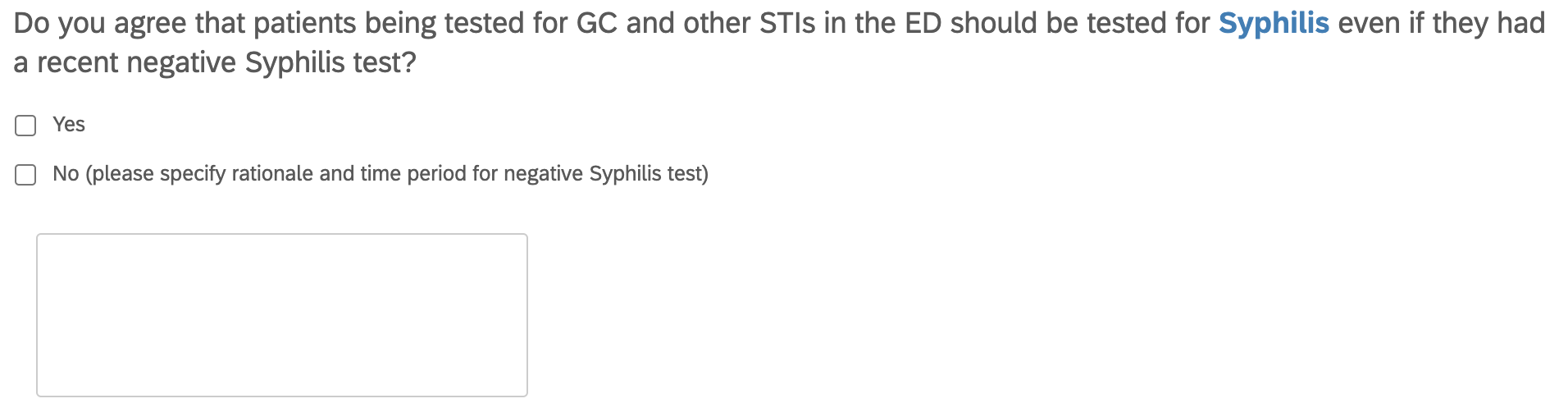


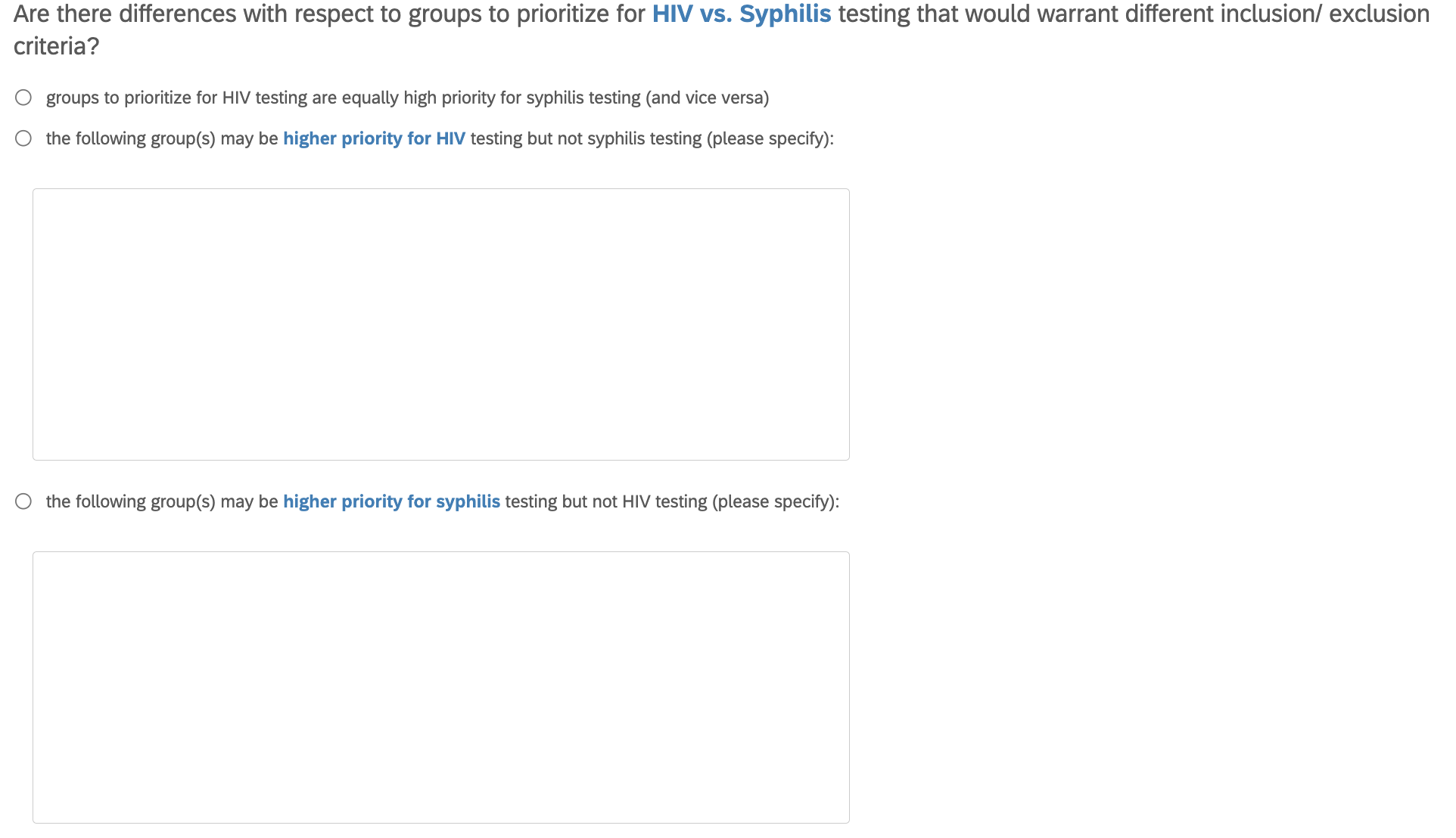


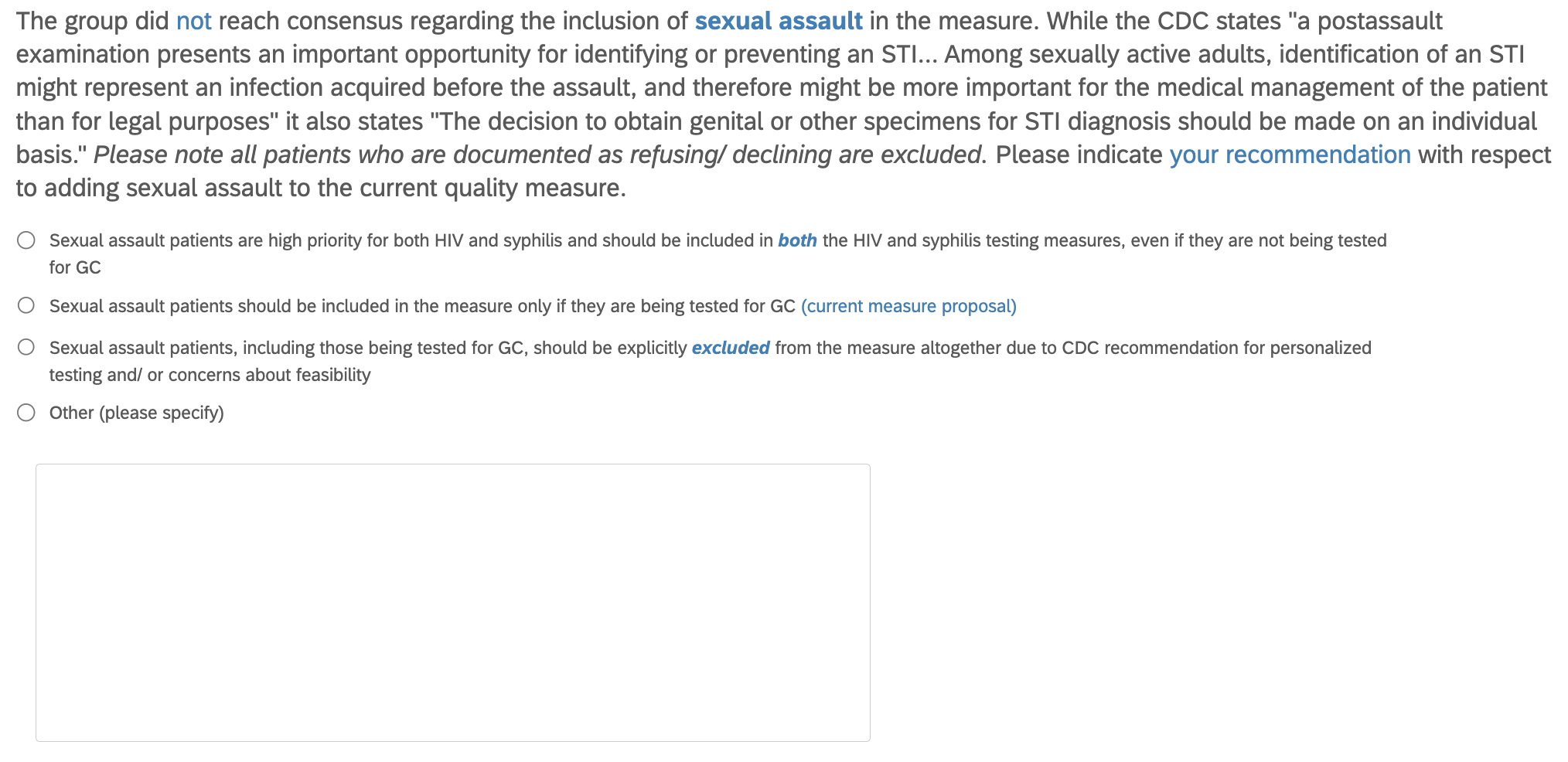


The group reached a consensus to **not** include the following high-impact groups in the current measure based on feasibility, with the intent to add them in the future when data can be captured more reliably (e.g. ICD-10, non-ED testing):

- Any recent STI
- Patients with a positive pregnancy test in the ED
- All pregnant patients regardless of reason for ED visit
- People who inject drugs
- High community prevalence
- Co-testing with HPV/Herpes/BV
- **All** patients admitted from ED, regardless of admitting diagnosis
- Men who have sex with men
- Persons in correctional facilities
- Recently incarcerated persons
- Unprotected sex with partner of unknown status

Please comment if you have any concerns about **not** including these groups in the measure at this time.

Do you have any general concerns that were not yet addressed? If so, please describe further below.
